# Summary of the best evidence for stress ulcer prophylaxis in critically ill patients

**DOI:** 10.3389/fmed.2026.1875134

**Published:** 2026-08-27

**Authors:** Jin Wang, Xiaoxia Tang, Linhui Jin, Yunxia Shen, Jiang Zhang, Jixiong Shi, Ting Yin, Guilan Zhang, Hailin Zhu

**Affiliations:** 1 Department of Nursing, Yunnan University of Traditional Chinese Medicine, Kunming, China; 2 Kunming Municipal Hospital of Traditional Chinese Medicine, Kunming, China; 3 Yunnan Cancer Hospital, Kunming, China; 4 Chongqing Hospital of the Affiliated Hospital of Guangzhou University of Chinese Medicine, Chongqing, China

**Keywords:** clinical practice, critically ill, evidence summary, evidence-based practice, stress ulcer prophylaxis

## Abstract

**Objective:**

To gather, assess, and integrate the most reliable evidence regarding stress ulcer prevention in critically ill individuals, thus offering guidance for clinical application.

**Methods:**

A comprehensive search was performed across various Chinese and English platforms and databases, such as UpToDate, National Guideline Clearinghouse (NGC), Registered Nurses' Association of Ontario (RNAO), Cumulative Index to Nursing and Allied Health Literature (CINAHL), Guidelines International Network (GIN), Eastern Association for the Surgery of Trauma (EAST), the Cochrane Library, PubMed, Web of Science, CNKI, Wangfang, VIP, Sinomed, the Full-text Database of Chinese Medical Journals, and Medlive. This search encompassed clinical decision-making tools, guidelines, systematic reviews, evidence summaries, expert consensus documents, randomized controlled trials (RCTs), and cohort studies. Additionally, a search for gray literature was carried out using Opengrey and DANS. The timeframe for the search extended from the establishment of each database up to April 14, 2026. Two researchers carried out independent assessments of literature quality, evidence extraction, and synthesis.

**Results:**

In total, 29 publications were analyzed, which included 1 clinical decision document, 6 guidelines, 4 expert consensus statements, 2 systematic reviews, 13 meta-analyses, 2 cohort studies, and 1 randomized controlled trial (RCT). Ultimately, 32 key evidence statements were compiled across seven categories: risk factors, indications for prophylaxis and decision-making, selection of agents and methods of administration, integrated decision-making concerning enteral nutrition and stress ulcer prophylaxis (SUP), possible harms and adverse events, diagnosis and monitoring, as well as special populations and scenarios. In summary, this research consolidates the most reliable information regarding the prevention of stress ulcers (SUs) in patients with critical illnesses. Healthcare providers can utilize this evidence in a tailored way, taking into account the specific clinical situation and the preferences of the patient. The results offer a solid evidence-based framework for standardizing clinical approaches and enhancing patient outcomes.

**Systematic review registration:**

The systematic review can be found at http://ebn.nursing.fudan.edu.cn/home, and its registration number is “ES202610400.”

## 1 Introduction

Stress ulcers (SUs) are prevalent acute lesions of the gastrointestinal mucosa found in critically ill patients. The occurrence of gastrointestinal bleeding due to SUs can greatly elevate the risk of mortality and extend the duration of ICU stays (1). Research indicates that the mortality rate for critically ill individuals experiencing significant gastrointestinal bleeding related to stress ulcers can soar to 49%, a stark contrast to the 9% seen in those without such bleeding (2). As a result, the implementation of stress ulcer prophylaxis (SUP) has become a standard procedure in intensive care settings. Nonetheless, there exists a paradox of both excessive and insufficient use of SUP in clinical environments. Evidence shows that between 68% and 79% of patients are prescribed acid-suppressive treatments without valid indications (3–5); Conversely, while medications like proton pump inhibitors (PPIs) can mitigate bleeding risks, they may also lead to negative outcomes such as hospital-acquired pneumonia, *Clostridioides difficile* infections, and kidney damage (6–8). Furthermore, both national and international guidelines on SUP present inconsistent or even contradictory advice regarding key aspects such as the appropriate patient population, medication choices, and treatment duration, with the overall quality of these guidelines being less than ideal (9). To address the disconnect between evidence and clinical practice, this study systematically gathered and evaluated current research on stress ulcer prophylaxis from both domestic and international sources, employing evidence-based approaches to assess, synthesize, and rank the findings. The goal is to equip healthcare professionals with clear, actionable guidance rooted in the best available evidence, thereby enhancing the standardized and personalized use of SUP.

## 2 Materials and methods

### 2.1 Criteria for summarizing evidence

This research followed the reporting guidelines established by the Fudan University Center for Evidence-Based Nursing, which encompassed defining the problem, gathering evidence, screening literature, assessing the quality of the studies, and synthesizing and grading the evidence (Registration No.: ES202610400). To improve the clarity and reproducibility of the research methodology, the study also complied with the PRISMA Statement, and a full PRISMA checklist was included in the Supplementary material to guarantee that the report is thorough, standardized, and verifiable. The processes of literature search, screening, quality evaluation, and evidence synthesis were carried out independently by two researchers, with any disagreements resolved through consensus or by consulting the corresponding author.

### 2.2 Establishment of the problem

Based on the PIPOST model, construct evidence-based questions:

P (Population): Adult critically ill patients (≥18 years) in the intensive care unit (ICU).

I (Intervention): Various forms of stress ulcer prophylaxis.

Healthcare experts engaged in the implementation of evidence, especially those in clinical roles, nursing, and other health-related fields.

O (Outcomes): Rates of major upper gastrointestinal hemorrhaging, occurrences of pneumonia acquired during hospitalization, frequency of *Clostridioides difficile* infections, mortality within 28 days, duration of stay in the ICU, and incidents of negative drug reactions, among others.

Locations: Critical care environments such as general intensive care units, specialized critical care units, emergency intensive care units, and other closely monitored healthcare settings.

Types of evidence include clinical guidelines, expert consensus statements, systematic reviews, meta-analyses, evidence summaries, randomized controlled trials (RCTs), and cohort studies.

### 2.3 Literature search strategy

A comprehensive search was performed across various platforms, including UpToDate, the National Guideline Clearinghouse (NGC), the Registered Nurses' Association of Ontario (RNAO), the Cumulative Index to Nursing and Allied Health Literature (CINAHL), the Guidelines International Network (GIN), the Eastern Association for the Surgery of Trauma (EAST), the Cochrane Library, the U.S. National Library of Medicine's PubMed, Web of Science, as well as CNKI, Wanfang Data, VIP, the China Biomedical Database, the Chinese Medical Journal Full-Text Database, and Medlive. Additionally, a search for gray literature was carried out using Opengrey and DANS. The timeframe for the search extended from the establishment of each database up to April 14, 2026. The English search terms included phrases such as “stress ulcer/gastric mucosal lesions/gastric mucosal erosion/gastric mucosa injury/stress-related mucosal disease/gastrointestinal bleeding/gastrointestinal hemorrhage,” “proton pump inhibitors/histamine H2 antagonists/sucralfate/enteral nutrition/antacids,” and “guideline/systematic review/meta-analysis/consensus/evidence,” among others, utilized in both subject headings and free-text queries. In Chinese, the search terms encompassed “stress ulcer/acute gastric mucosal lesions/acute gastric mucosal erosion/acute gastric mucosal injury/stress-related mucosal lesions/upper gastrointestinal bleeding,” “prevention/prevention strategies/pharmacological prevention/proton pump inhibitors/omeprazole/pantoprazole/H2 receptor antagonists (H2RAs)/ranitidine/famotidine/sucralfate/enteral nutrition,” and “guidelines/expert consensus/systematic review/meta-analysis/evidence summary.” The search strategy for PubMed is detailed in Table 1, while the approaches for the other databases can be found in the Supplementary material.

**Table 1:** PubMed search strategy.

Search	Query
#1	“stress ulcer”[Title/Abstract] OR “gastric mucosal lesions”[Title/Abstract] OR “gastric mucosal erosion”[Title/Abstract] OR “gastric mucosa injury”[Title/Abstract] OR “stress related mucosal disease”[Title/Abstract] OR “gastrointestinal bleeding”[Title/Abstract] OR “gastrointestinal hemorrhage”[Title/Abstract]
#2	“proton pump inhibitors”[Pharmacological Action] OR “proton pump inhibitors”[Supplementary Concept] OR “proton pump inhibitors”[All Fields] OR “proton pump inhibitors”[MeSH Terms] OR (“proton”[All Fields] AND “pump”[All Fields] AND “inhibitors”[All Fields]) OR (“histamine h2 antagonists”[Pharmacological Action] OR “histamine h2 antagonists”[Supplementary Concept] OR “histamine h2 antagonists”[All Fields] OR “histamine h2 antagonists”[MeSH Terms] OR (“histamine”[All Fields] AND “h2”[All Fields] AND “antagonists”[All Fields])) OR (“sucralfate”[Supplementary Concept] OR “sucralfate”[All Fields] OR “sucralfate”[MeSH Terms]) OR (“enteral nutrition”[MeSH Terms] OR (“enteral”[All Fields] AND “nutrition”[All Fields]) OR “enteral nutrition”[All Fields]) OR (“antacidic”[All Fields] OR “antacids”[Pharmacological Action] OR “antacids”[Supplementary Concept] OR “antacids”[All Fields] OR “antacid”[All Fields] OR “antacids”[MeSH Terms])
#3	“guideline”[Publication Type] OR “guidelines as topic”[MeSH Terms] OR “guideline”[All Fields] OR ((“expert”[All Fields] OR “expert s”[All Fields] OR “expertize”[All Fields] OR “experts”[All Fields]) AND (“consensual”[All Fields] OR “consensually”[All Fields] OR “consensus”[MeSH Terms] OR “consensus”[All Fields])) OR (“systematic review”[Publication Type] OR “systematic reviews as topic”[MeSH Terms] OR “systematic review”[All Fields]) OR (“meta analysis”[Publication Type] OR “meta analysis as topic”[MeSH Terms] OR “meta analysis”[All Fields]) OR ((“evidence”[All Fields] OR “evidences”[All Fields] OR “evident”[All Fields] OR “evidently”[All Fields]) AND (“summaries”[All Fields] OR “summary”[All Fields])) OR (“recommend”[All Fields] OR “recommendable”[All Fields] OR “recommendation”[All Fields] OR “recommendation s”[All Fields] OR “recommendations”[All Fields] OR “recommended”[All Fields] OR “recommending”[All Fields] OR “recommends”[All Fields])
#4	#1 AND #2 AND #3

### 2.4 Criteria for considering studies

The criteria for inclusion included: (i) patients aged 18 and older who are in critical condition within the ICU; and (ii) information pertaining to methods aimed at preventing stress ulcers.

The criteria for exclusion included: (i) publications lacking complete data or full text access; (ii) articles not in Chinese or English; (iii) abstracts or conference proceedings; and (iv) documents that interpret guidelines.

### 2.5 Literature screening

Two researchers conducted literature searches, evaluations, and quality assessments separately. The records obtained at first were uploaded into Endnote reference management software to eliminate duplicates. The titles and abstracts were reviewed for preliminary screening. Following this initial review, the full texts of the articles that passed the first screening were examined for a secondary evaluation, leading to the selection of the final literature.

### 2.6 Evaluation of literature quality

The evaluation of the guidelines was conducted using the Appraisal of Guidelines for Research and Evaluation (AGREE II) (10, 11). This framework includes six domains and a total of 23 items, each rated on a scale from one to seven, where one indicates \strongly disagree\ and seven signifies \strongly agree.\ The standardized percentage score for each domain is calculated as follows: (total score from all reviewers – minimum possible score)/(maximum possible score – minimum possible score) × 100%. The minimum score for each domain is determined by multiplying 1 point by the number of items and the number of evaluators, while the maximum score is calculated by multiplying seven points by the same factors. A higher percentage score reflects superior guideline quality. Recommendations are categorized into three levels: Grade A is given when all six domains have scores exceeding 60%; Grade B is assigned when three or more domains score between 30% and 60%; and Grade C is for scores below 30% in three or more domains. The quality of the clinical decision aids was evaluated using the Critical Appraisal for Summaries of Evidence (CASE) tool (12). Additionally, expert consensus statements, systematic reviews, meta-analyses, and randomized controlled trials (RCTs) were assessed based on the criteria from the Authenticity Assessment Tool (2016 edition) by the Australian JBI Center for Evidence-Based Healthcare (13). Due to the potential variability among the study types included, which precluded the use of funnel plots and Egger's test, a supplementary search for gray literature was performed alongside the primary databases to reduce the effects of publication bias and selective reporting as much as possible.

### 2.7 Evidence synthesis

Following the independent extraction of evidence by two researchers, they proceeded to verify their results against each other. The review incorporated a diverse range of study designs, including guidelines, expert consensus statements, systematic reviews, cohort studies, and randomized controlled trials. To synthesize the evidence, the researchers implemented the following strategies: (1) Hierarchical synthesis principle: Utilizing the JBI evidence pre-classification and the 2014 edition of the JBI evidence recommendation grading system (14), where Level 1a represents the highest quality and Level 5c the lowest. For each clinical inquiry, the highest quality evidence was prioritized, while lower-level evidence was used as additional support when high-level evidence was adequate to address the question. (2) Guidelines for resolving conflicting evidence: In cases of discrepancies among sources, the following priorities were established: emphasis on the quality of study design; preference for the most recent evidence; and consideration of sample size and precision. After grading the evidence, the research team utilized the JBI FAME framework to evaluate the feasibility, appropriateness, relevance, and validity of each piece of evidence, assigning a recommendation grade of A (strong recommendation) or B (weak recommendation) to ensure a systematic and consistent evaluation process (15–17). Any differences in opinion were settled through consensus or by consulting the corresponding author.

In order to assist healthcare professionals in comprehending the clinical significance of the recommendation grades, the research group established the following definitions for the JBI recommendation grades: Grade A (Strong Recommendation): The advantages of following this recommendation greatly surpass the associated risks, indicating that it should be adhered to in most clinical scenarios. Grade B (Weak Recommendation): While there is some supporting evidence, there remains significant uncertainty about the trade-off between benefits and risks. Clinicians are encouraged to make tailored decisions that consider the unique circumstances of each patient.

## 3 Results

### 3.1 Search results

**Figure 1:**
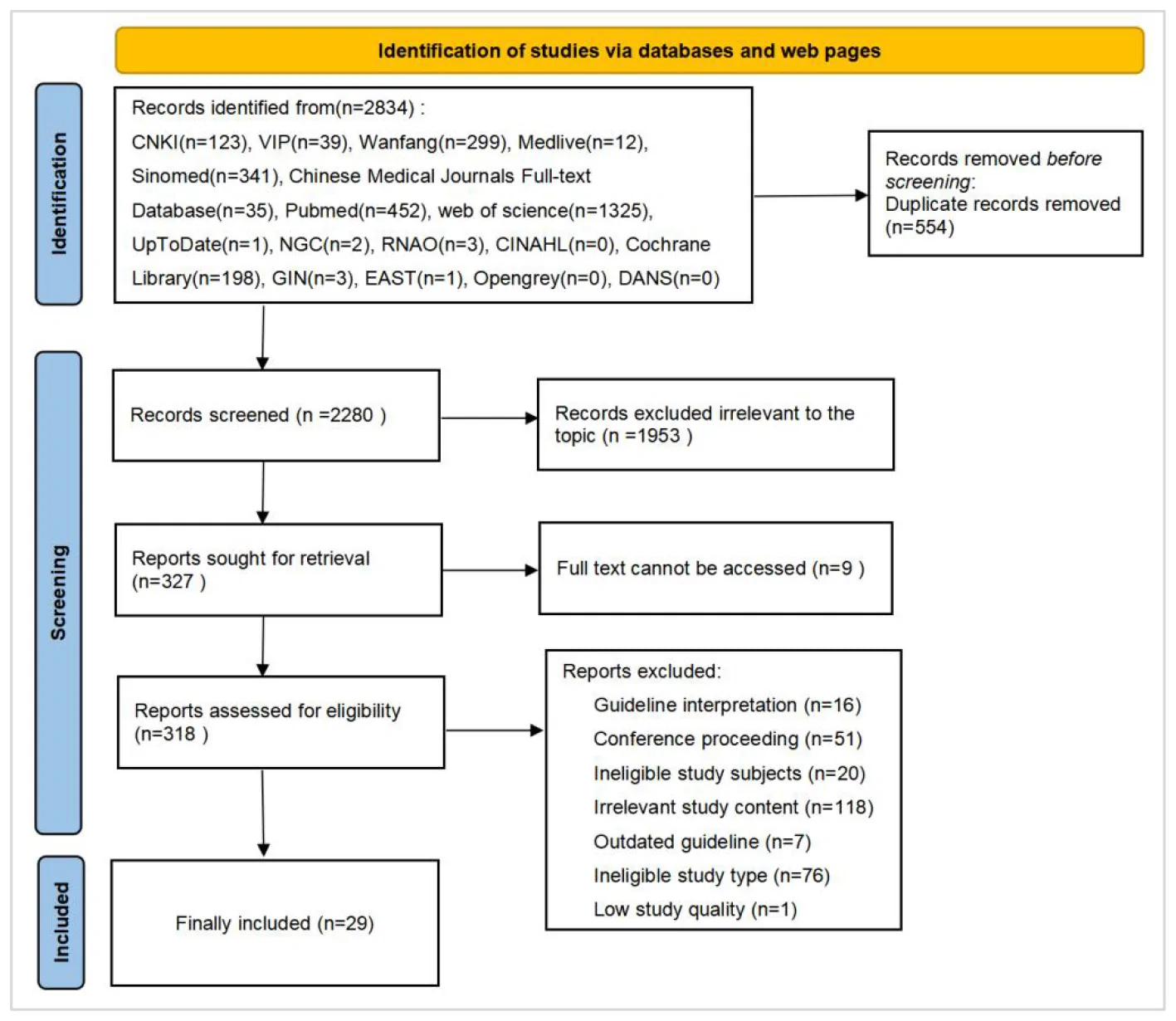
Literature screening flow diagram.

An initial search uncovered 2,834 records. After implementing a multi-step screening procedure, 29 studies were determined to fulfill the eligibility requirements for final inclusion. The process of selecting studies is illustrated in Figure 1.

### 3.2 Overview of included literature

A total of 29 publications were incorporated into this research. This collection comprised one study focused on clinical decision-making (18); six sets of guidelines (19–24); four documents reflecting expert consensus (25–28); two systematic reviews (9, 29); 13 meta-analyses (30–42); 2 cohort studies (43, 44); and one randomized controlled trial (RCT) (45). Table 2 outlines the overall features of these studies.

**Table 2:** Literature general information.

Reference	Country	Literature source	Literature type	Literature topic
Weinhouse (18)	USA	UpToDate	Clinical decision	Diagnosis, Treatment, and Prevention of Stress Ulcer
Dechang (19)	China	Chinese medical journals full-text database	Guideline	Prevention, diagnosis, and treatment of stress ulcers in critically ill patients
Amer et al. (21)	Saudi Arabia	Pubmed	Guideline	Contextualized clinical practice guideline for stress ulcer prophylaxis in critically ill adults
Ye et al. (22)	International	Pubmed	Guideline	Rapid clinical practice guideline for gastrointestinal bleeding prophylaxis in critically ill patients
MacLaren et al. (23)	USA	Pubmed	Guideline	Prevention of stress-related gastrointestinal bleeding in critically ill adults
Lin (20)	China	Web of science	Guideline	Appropriate use of gastric acid suppressants in gastrointestinal surgery
EAST Practice Management Guidelines Committee (24)	USA	EAST	Guideline	Practice management guidelines for stress ulcer prophylaxis in trauma patients
Jiyao (26)	China	Chinese medical journals full-text database	Expert consensus	Prevention and treatment of stress ulcer after traumatic brain injury
Shudong et al. (28)	China	Medlive	Expert consensus	Definition, etiology, diagnosis, prevention and treatment of stress ulcer
Yu et al. (27)	China	Chinese medical journals full-text database	Expert consensus	Prevention and treatment strategies for stress ulcer in critically ill patients
Yupei (25)	China	Medlive	Expert consensus	Prevention and treatment of stress-related mucosal disease in general surgery patients
Nourian et al. (45)	Iran	Pubmed	RCT	Comparison of enteral nutrition plus ranitidine vs. enteral nutrition alone for stress ulcer prophylaxis
Zhiqiao et al. (9)	China	Wanfang	Systematic review	Methodological quality assessment and comparison of recommendations in stress ulcer prophylaxis guidelines
Krag et al. (29)	Denmark	Pubmed	Systematic review	Indications and evidence status for stress ulcer prophylaxis in ICU patients
Huang et al. (43)	China	Pubmed	Retrospective cohort study	Comparison of PPI vs. H2RA for stress ulcer prophylaxis in septic patients (based on MIMIC-III database)
Song et al. (44)	South Korea	web of science	Retrospective cohort study	Comparison of PPI vs. H2RA for stress ulcer prophylaxis in ICU patients
Junfeng et al. (32)	China	VIP	Meta-analysis	Comparison of PPI vs. H2RA for prevention of stress ulcer bleeding and pneumonia in ICU patients
Zhongfang et al. (33)	China	Chinese medical journals full-text database	Meta-analysis	Efficacy of pantoprazole vs. H2RA for stress ulcer bleeding in intracerebral hemorrhage patients
Alhazzani et al. (30)	Canada	Pubmed	Meta-analysis	Comparison of PPI vs. H2RA for stress ulcer prophylaxis in critically ill patients
Liu et al. (31)	China	Pubmed	Meta-analysis	Risks and benefits of stress ulcer prophylaxis in neurocritical care patients
Alshamsi et al. (34)	Canada	Pubmed	Meta-analysis	Efficacy and safety of PPI vs. H2RA for stress ulcer prophylaxis
Alhazzani et al. (35)	Canada	Pubmed	Meta-analysis	Network meta-analysis comparing PPI, H2RA, sucralfate, and placebo
Wang et al. (37)	China	Pubmed	Meta-analysis	Updated network meta-analysis including the PEPTIC trial
Wang et al. (36)	China	Pubmed	Meta-analysis	Network meta-analysis of gastrointestinal bleeding prophylaxis in critically ill patients
Huang et al. (38)	China	Pubmed	Meta-analysis	Whether pharmacological stress ulcer prophylaxis is needed in ICU patients receiving enteral nutrition
Sridharan et al. (39)	USA	Pubmed	Meta-analysis	Network meta-analysis of multiple pharmacological interventions for stress ulcer prophylaxis
Barbateskovic et al. (40)	Denmark	Pubmed	Meta-analysis	Comparison of PPI or H2RA vs. placebo/no prophylaxis in ICU patients
Deliwala et al. (41)	USA	Pubmed	Meta-analysis	Efficacy and safety of PPI vs. H2RA for stress ulcer prophylaxis in critically ill patients
Marik et al. (42)	USA	Pubmed	Meta-analysis	Impact of enteral nutrition on the efficacy of H2RA for stress ulcer prophylaxis

### 3.3 Literature quality evaluation results

Among the 29 studies reviewed, one served as a clinical decision aid. With the exception of Question 3, which did not disclose the names of the reviewers, all other questions received a “Yes” evaluation. There were six guidelines, five of which were assigned an A recommendation grade, while one received a B; the comprehensive quality assessment findings are presented in Table 3. Additionally, four expert consensus documents were part of the review. The research conducted by Xiao Shudong and colleagues was rated “No” for criterion four, which assesses whether the views are grounded in significant research literature and include citations, and received an “Unclear” rating for criterion five, which evaluates the balance between literature evidence and expert opinion. All other studies achieved “Yes” ratings across all criteria. Both systematic reviews included were rated “Yes” for every criterion. The sole randomized controlled trial (RCT) included did not implement blinding and failed to conduct an intention-to-treat analysis for all participants; nevertheless, its objectives were clearly defined, and the results were appropriate for evidence synthesis. The two cohort studies were marked as “Not applicable” regarding follow-up loss criteria but satisfied all other requirements. All 13 meta-analyses included received “Yes” ratings for every criterion and exhibited relatively thorough study designs. The overall quality of the literature reviewed was high, leading to the inclusion of all studies.

**Table 3:** Methodological quality assessment of included guidelines.

Reference	Standardized percentage scores by domain (%)						Number of domains (≥60%)	Number of domains (< 30%)	Grade
	Scope and purpose	Stakeholder involvement	Rigor of development	Clarity of presentation	Applicability	Editorial independence			
Dechang (19)	91.0	80.0	92.3	89.7	80.0	90.0	6	0	A
Amer et al. (21)	88.3	85.0	90.8	84.0	80.0	90.0	6	0	A
Ye et al. (22)	87.2	85.0	80.0	85.0	70.0	90.0	6	0	A
MacLaren et al. (23)	84.3	82.4	92.7	87.3	82.1	91.0	6	0	A
Lin (20)	76.8	80.1	85.0	85.4	75.1	80.0	6	0	A
EAST Practice Management Guidelines Committee (24)	68.0	55.3	48.3	70.3	54.0	60.1	3	0	B

### 3.4 Summary and grading of evidence

Data was gathered, analyzed, and evaluated from the studies that were part of the review. In total, 32 key findings were compiled across seven categories: risk elements; guidelines for prevention and decision processes; choices of medication and methods of delivery; integrated decision-making for enteral nutrition and stress ulcer prophylaxis; possible risks and negative outcomes; assessment and tracking; as well as unique groups and situations. Refer to Table 4 for further information.

**Table 4:** Best evidence summary for stress ulcer prophylaxis in critically ill patients.

Evidence domain	Evidence statement	Level	Grade
Risk factors	1. Major/primary risk factors for stress ulcer and clinically important upper gastrointestinal bleeding in critically ill patients include: coagulopathy (PLT < 50,000/μl, INR >1.5, or PTT >2 times normal), shock, chronic liver disease (cirrhosis, portal hypertension), and mechanical ventilation >48 h (9, 17–23, 25)	1a	A
	2. Other risk factors include: sepsis, renal replacement therapy, acute kidney failure, ARDS, severe traumatic brain/spinal cord injury, burns >30%, severe trauma/polytrauma, major complex surgery, history of peptic ulcer/GI bleeding within 1 year, high-dose corticosteroids (hydrocortisone >250 mg/day), ICU stay >1 week, fecal occult blood for ≥3 days, use of anticoagulants/antiplatelets/NSAIDs, organ transplantation, severe jaundice, cerebrovascular accident, severe psychological stress, male sex, advanced age (≥65 years) (9, 17–23, 25–27)	1b	A
	3. Presence of any two or more of the following secondary risk factors significantly increases bleeding risk: sepsis, ICU stay >1 week, fecal occult blood for >3 days, high-dose corticosteroids, use of NSAIDs or antiplatelet agents (19, 25–27)	2b	B
Indications for prophylaxis	4. For patients with any one of coagulopathy, shock, or chronic liver disease, SUP is strongly recommended (17–23)	1a	A
	5. For patients receiving mechanical ventilation >48 h (especially without enteral nutrition), SUP is recommended (9, 17, 19, 21, 23, 25)	1a	A
	6. For patients in neuro-intensive care (with traumatic brain or spinal cord injuries), the use of SUP may be considered to reduce the risk of upper gastrointestinal bleeding. However, as the evidence is primarily based on expert consensus, the patient's risk of infection should also be assessed when making clinical decisions (22, 25, 30)	2b	B
	7. For low-risk patients without any risk factor, routine SUP is not recommended (18, 21, 22)	1b	A
	8. For patients in whom risk factors for bleeding have been eliminated (e.g., those who have been weaned off the ventilator and have normal coagulation function), it is recommended that the need for continued SUP use be reassessed promptly, and that discontinuation be considered at the appropriate time (20, 22)	2b	B
Agent selection and mode of administration	9. PPIs or H2RAs are recommended as first-line prophylactic agents, rather than sucralfate, antacids, or enteral nutrition alone (9, 17, 18, 20, 22, 23, 25–27, 44)	1a	A
	10. For prevention of clinically important upper GI bleeding, PPIs appear to be more effective than H2RAs. However, this benefit must be weighed against the potential for increased pneumonia risk and possible mortality signals in specific subgroups (17, 21, 26, 29, 31, 33–36, 40)	1a	A
	11. PPIs and H2RAs show no significant difference in mortality, although some studies suggest PPIs may slightly increase mortality risk in specific populations (e.g., sepsis, high disease severity) (21, 35, 36, 39, 42, 43)	1a	B
	12. Both PPIs and H2RAs are effective in preventing overt upper GI bleeding and are superior to placebo/no prophylaxis (29–34, 38)	1a	A
	13. Sucralfate may be an alternative when PPIs or H2RAs are not available; antacids are no longer recommended (17, 22, 23, 25, 27)	2b	B
	14. In septic patients, current evidence suggests PPI may increase hospital mortality, GI bleeding, and pneumonia risk; caution is advised (42)	2b	B
	15. Aluminum-containing agents (e.g., sucralfate, aluminum hydroxide) should be avoided in dialysis patients to prevent aluminum toxicity (23)	2b	B
	16. For patients able to receive enteral medication, oral/nasogastric PPI is preferred; for those unable to receive enteral medication, intravenous PPI or H2RA may be given (17, 20, 22)	2b	B
	17. Low-dose PPI or H2RA regimens are recommended rather than high-dose regimens (20, 22)	2b	B
	18. In patients requiring long-term (≥3 months) PPI use, potential risks (infection, fracture, kidney disease) should be assessed, and high-risk patients monitored accordingly (19, 25, 26)	2a	B
	19. SUP should be stopped when risk factors have resolved, the patient is stable, can tolerate enteral nutrition, or is discharged from the ICU (9, 17, 20, 22, 25, 26)	2b	B
Enteral nutrition and SUP	20. Early enteral nutrition helps maintain gastrointestinal mucosal integrity and may reduce stress ulcer risk. In low-risk patients receiving enteral nutrition, routine pharmacological SUP is not recommended (18, 20–22, 25, 26, 37, 41, 44)	1a	A
	21. In patients receiving enteral nutrition but with persistent risk factors, pharmacological SUP should be continued (17, 20, 22)	2a	B
	22. Enteral nutrition alone (without medication) may be insufficient to prevent stress ulcers in high-risk patients (23, 44)	2b	B
	23. If bloody gastric contents < 100 ml appear during enteral nutrition, feeding may be continued with addition of a PPI; if >100 ml, enteral nutrition should be used with caution (25)	4b	B
Potential harms and adverse events	24. PPIs and H2RAs may increase the risk of hospital-acquired pneumonia, but this should not preclude SUP in high-risk patients (17, 18, 21, 27, 34–38, 41)	1a	A
	25. Long-term PPI use (≥3 months) requires attention to risk of *Clostridioides difficile* infection, kidney adverse events, fracture, and stroke (17–20, 22, 25, 26)	2a	B
	26. The effect of PPIs and H2RAs on *C. difficile* infection remains uncertain; current data are weak and conflicting (17, 21, 31, 43)	2b	B
Diagnosis and monitoring	27. Clinical presentation of stress ulcers: often without prodromal symptoms (epigastric pain, acid reflux); mainly manifest as upper GI bleeding (hematemesis, melena), unexplained hemoglobin drop (≥20 g/L), or hemorrhagic shock; perforation may present as acute abdomen (17, 19, 25–27)	3b	B
	28. Diagnostic assessment: in cases of occult bleeding, follow-up should be carried out to confirm remission; in cases of overt bleeding, endoscopy may be performed; endoscopy is recommended only in cases of major hemorrhage where medical treatment has proved ineffective, and is not recommended as a routine method of early diagnosis (17–19)	3b	B
	29. Monitoring measures: intermittent monitoring of gastric pH and fecal occult blood testing may be considered as supplementary monitoring methods; however, their direct value in improving prognosis remains unclear, and the duration of SUP treatment should not be unduly prolonged on the basis of these monitoring results (19, 25, 26)	4c	B
Special populations and scenarios	30. Neurocritical care patients (traumatic brain/spinal cord injury): SUP reduces upper GI bleeding and all-cause mortality without increasing nosocomial pneumonia risk (25, 30)	1a	A
	31. Perioperative patients: for patients undergoing major surgery who are at high risk of developing stress ulcers postoperatively, oral acid suppressants may be administered during the perioperative period as appropriate (9, 25, 27)	2b	B
	32. Organ transplant recipients: as this group is considered high-risk, clinicians should monitor them for the risk of stress ulcers and carry out an individualized assessment to determine whether SUP is required (19, 26, 27)	3b	B

Evidence level: according to the JBI evidence pre-grading system. Level 1 is the highest, Level 5 the lowest. Grade of recommendation: Grade A = strong recommendation, Grade B = weak recommendation. The recommendation grade is determined based on the strength of the evidence and the clinical benefit-to-risk ratio. SUP, stress ulcer prophylaxis; PLT, platelet; INR, international standard ratio; PPT, partial thromboplastin time. Clinically important upper GI bleeding: bleeding with hemodynamic changes or need for transfusion. PPI, proton pump inhibitor; H2RA, histamine-2 receptor antagonist.

### 3.5 SUP clinical decision pathway

**Figure 2:**
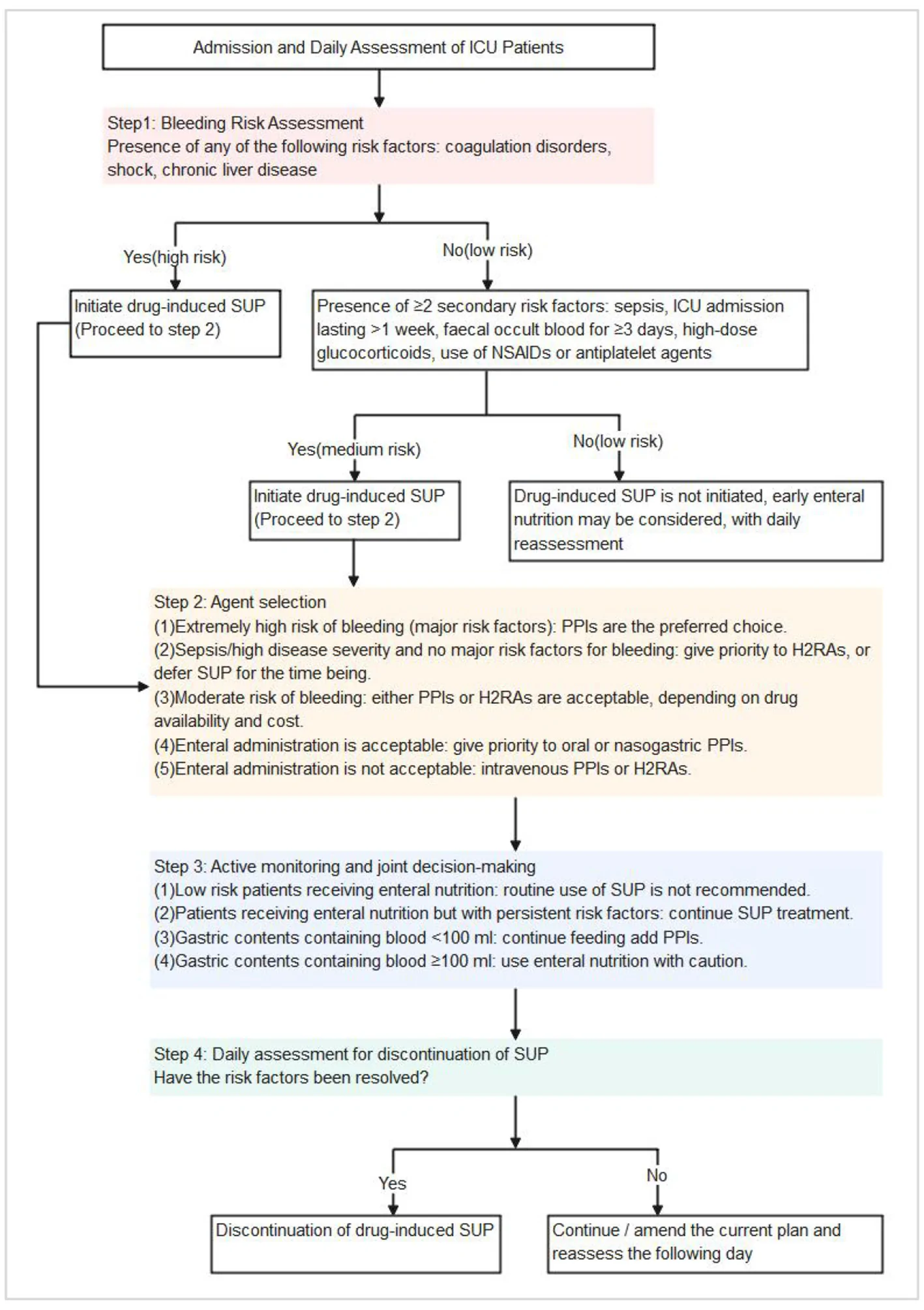
Clinical decision pathway for stress ulcer prophylaxis in critically ill patients.

In order to assist healthcare practitioners in swiftly utilizing the aforementioned evidence, the research group created a clinical decision framework aimed at preventing stress ulcers in patients with critical conditions, drawing from a synthesis of 32 evidence sources (see Figure 2).

## 4 Discussion

### 4.1 Risk stratification and integration of evidence into early decision-making

The successful application of SUP hinges on the precise identification of patients who will genuinely gain from preventive measures. This research highlights that significant independent risk factors for clinically relevant upper gastrointestinal bleeding include coagulation disorders, shock, chronic liver disease, and extended mechanical ventilation (over 48 h). Importantly, the findings differentiate between \major\ and \minor\ risk factors, suggesting that having any two minor factors considerably heightens the risk of bleeding. This approach to risk stratification resembles scoring systems utilized in cardiology and venous thromboembolism, yet it is not commonly adopted in standard ICU practices. A potential explanation for this underutilization is the absence of a standardized, prospectively validated SUP risk assessment that can be applied across various ICU settings. To enhance clinical practice, we advise that ICUs perform risk evaluations within the first 24 h of patient admission, utilizing a checklist that incorporates the four primary risk factors and the composite criteria for secondary factors. This method will effectively minimize unnecessary SUP administration in patients at low risk while ensuring that those at high risk receive prompt treatment.

### 4.2 Drug selection: efficacy, mortality, and unresolved controversies

This research highlights a complex dilemma in drug choice. While proton pump inhibitors (PPIs) are more effective than H2 receptor antagonists (H2RAs) in reducing significant upper gastrointestinal bleeding, as supported by various meta-analyses, this advantage does not extend to overall mortality rates, where no notable difference exists between the two classes of drugs. More troubling is the indication that PPIs might be linked to higher mortality in certain high-risk groups, especially among patients with severe conditions like sepsis. This is not a novel discovery; retrospective analyses from numerous large-scale observational studies and randomized trials have shown that critically ill individuals on PPIs may experience increased mortality within 90 days. Possible explanations for this include unintended immunosuppression, changes in gut microbiota, or heightened vulnerability to hospital-acquired infections. Although current data do not warrant a blanket avoidance of PPIs in all sepsis patients, it does suggest a need for careful consideration. Consequently, we advocate for a personalized, phenotype-driven approach to drug selection: PPIs should be the preferred option for those at very high bleeding risk, with vigilant monitoring for secondary infections; for sepsis patients or those with severe disease scores lacking significant bleeding risk factors, H2RAs might be a better choice, or pharmacological stress ulcer prophylaxis (SUP) could be postponed if early enteral nutrition is possible; and for individuals at moderate bleeding risk, either medication can be chosen based on a thorough evaluation of drug availability, cost, and specific patient circumstances.

### 4.3 Enteral nutrition as a modifying factor for the benefits and risks of SUP

The relationship between enteral nutrition and stress ulcer prophylaxis (SUP) is frequently neglected, yet it carries important clinical consequences. This summary of evidence suggests that initiating enteral nutrition early can mitigate mucosal damage caused by stress. For patients deemed low-risk who are receiving sufficient enteral nutrition, the routine use of pharmacological SUP is not advised. Conversely, for those at high risk, relying solely on enteral nutrition does not adequately prevent stress ulcers. This creates a complex decision-making process: when considering SUP, both the risk of bleeding and the patient's nutritional condition should be evaluated together, rather than following a strict binary approach. For instance, a patient with bleeding disorders on mechanical ventilation and total enteral nutrition should still be given pharmacological SUP, while another patient who is only on mechanical ventilation, well-nourished, and without bleeding disorders might be monitored without medication. The evidence also offers practical recommendations for managing enteral nutrition during episodes of active bleeding: if the volume of bloody gastric output is less than 100 ml, feeding can proceed with a proton pump inhibitor; however, if it exceeds 100 ml, caution is advised with enteral nutrition. Although this guidance is based on lower-quality evidence, it addresses a frequent challenge in clinical settings. Future high-quality observational studies or practical trials are needed to confirm these thresholds and explore whether intermittent feeding vs. continuous feeding impacts bleeding risk.

### 4.4 Risks of SUP: balancing bleeding prevention against increased infection risk

A persistent issue associated with stress ulcer prophylaxis (SUP) is the heightened likelihood of developing pneumonia acquired in hospitals, which is thought to occur due to bacterial proliferation linked to elevated gastric pH levels. This overview indicates that both proton pump inhibitors (PPIs) and H2 receptor antagonists (H2RAs) contribute to this increased risk of hospital-acquired pneumonia. Nevertheless, the findings suggest that this risk should not deter the continuation of SUP in patients identified as having significant bleeding risk factors. Beyond pneumonia, prolonged use of PPIs (for 3 months or more) raises alarms about the potential for *Clostridioides difficile* infections, kidney-related complications, fractures, and an elevated risk of stroke. While most critically ill patients are typically given short-term SUP, certain individuals—particularly those with extended stays in intensive care units (ICUs) or multiple hospital admissions—might experience considerable cumulative exposure. The data concerning *Clostridioides difficile* infections is inconsistent and weak, likely influenced by confounding variables such as the severity of pre-existing conditions, antibiotic administration, and the effectiveness of infection control practices at healthcare facilities. Practically, we advise that ICUs conduct daily evaluations of the necessity for SUP and initiate automatic discontinuation procedures once the risk factors have been addressed.

### 4.5 Economic considerations and feasibility of implementation

While this research did not perform a standalone economic assessment, the current body of literature sheds light on the cost-effectiveness of stress ulcer prophylaxis (SUP) strategies. Pharmacoeconomic evaluations reveal that the cost-effectiveness of various SUP approaches differs based on the assumptions and data utilized in studies, leading to inconsistent findings. One cost-effectiveness analysis utilizing a decision tree model indicated that for high-risk patients, the average expense per uncomplicated case for the PPI regimen was $58,700, which is lower than the $63,920 associated with H2RA, implying that PPIs may be more cost-effective in preventing stress ulcer bleeding (46). Conversely, another study employing a decision analysis model found that the anticipated cost for the H2RA regimen was $9,039, with a complication rate of 17.6% and a mortality rate of 2.50%, while the PPI regimen had an expected cost of $11,249, a complication rate of 22.0%, and a mortality rate of 3.34%, suggesting that H2RAs could be more advantageous in terms of cost and outcomes (47). Importantly, a recent cost-effectiveness analysis derived from international multicenter data from the REVISE trial indicated that pantoprazole use for SUP in ICU patients on invasive mechanical ventilation not only effectively prevents upper gastrointestinal bleeding but also results in net healthcare savings by reducing ICU stay duration. This insight is crucial for clinical decision-making in settings with limited resources (48). In conclusion, while the economic evidence surrounding SUP is not definitive, available data imply that targeted SUP, when applied after accurately identifying high-risk individuals, may yield both clinical advantages and cost-effectiveness. In resource-limited environments, enhancing adherence to guidelines, increasing the role of clinical pharmacists, and creating a responsive mechanism for evaluating drug discontinuation are essential strategies for improving the cost-effectiveness of SUP.

### 4.6 Special populations and evidence gaps

This summary of evidence examines three distinct subgroups within the ICU: individuals suffering from severe neurological disorders (Evidence 30), patients undergoing surgery (Evidence 31), and those who have received organ transplants (Evidence 32). Given their particular pathophysiological conditions, the likelihood of developing stress ulcers and the risk-benefit analysis of stress ulcer prophylaxis (SUP) in these groups may vary from that of the broader patient population. While this summary has made an effort to categorize these unique groups and offer tailored recommendations, it is important to recognize that the studies referenced still show variability in patient selection criteria, severity of illness evaluations, existing health issues, and treatment protocols. Consequently, there is some ambiguity regarding how well these recommendations can be generalized to various ICU subgroups; healthcare providers should tailor their decisions to the specific context of their institution when considering these guidelines for individual patients. Additionally, data on highly specialized patient groups—such as those with acute liver failure or those on extracorporeal membrane oxygenation (ECMO)—is notably limited. This document cannot furnish specific guidance for these populations, highlighting the urgent need for further research in this area.

## 5 Summary

This research compiles the most reliable data on preventing stress ulcers in critically ill individuals, encompassing 32 pieces of evidence across seven categories. This information aims to offer evidence-based recommendations for healthcare practitioners in clinical settings. Nonetheless, certain limitations are present.

This research has notable limitations concerning the management of publication bias. As a quantitative meta-analysis of the original studies of the same category was not conducted, it was impossible to evaluate the extent of publication bias in the literature using statistical techniques like funnel plots or Egger's regression. While the search strategy incorporated gray literature to include more studies with negative outcomes, it could not entirely eliminate two specific bias risks: A publication bias that favors positive findings, potentially resulting in a systematic inflation of effect sizes regarding the preventive effectiveness of PPIs or H2RAs; and A selective reporting bias that might distort the benefit-risk assessment for certain recommendations in this synthesis, leaning toward an “excessive benefit” perspective. Future investigations should prioritize the open dissemination of negative findings and advocate for the registration and public availability of prospective studies to significantly mitigate the influence of publication bias on systematic reviews.

Additionally, a significant portion of the data supporting the advantages of low-dose PPIs and the protective role of enteral nutrition alternatives is derived from secondary analyses, with a scarcity of robust RCTs that make direct comparisons. Future studies should aim to identify the protective threshold of enteral nutrition to offer quantitative insights for strategies utilizing nutritional substitutes.

Due to the research team's limited ability to access, translate, and extract information from literature not in Chinese or English, the studies included were mainly published in these two languages. While this is a standard practice in evidence-based research, it does raise concerns about potential language bias. Most high-quality original research in critical care medicine is predominantly found in English journals, and incorporating Chinese literature is essential to accurately represent the regional aspects of domestic clinical practices. This strategy aims to strike a balance between the reliability of the evidence and the practical constraints of available resources. However, when applying this evidence to regions or countries where Chinese or English is not spoken, careful consideration of its external validity is necessary. Future studies might benefit from forming multilingual teams or using professional translation services to perform cross-national database searches, which could help to further validate and broaden the findings of this research.

Lastly, while this research referenced findings from prior pharmacoeconomic studies on SUP in its discussion, it fell short of performing an independent cost-effectiveness analysis and did not evaluate the accessibility and affordability of medications across various economic contexts. Subsequent studies should focus on localized economic assessments that consider medication costs, ICU resource distribution, and disease specifics in particular countries and areas to inform clinical choices in settings with limited resources.

## Data Availability

The original contributions presented in the study are included in the article/Supplementary material, further inquiries can be directed to the corresponding author/s.
