# Supplementary material for: Summary of the best evidence for stress ulcer prophylaxis in critically ill patients
Source: Front Med (Lausanne). 2026 Aug 27;13:1875134. doi: 10.3389/fmed.2026.1875134 (PMC13558107; doi:10.3389/fmed.2026.1875134)
Supplement: Supplementary file 2 [file Table_2.docx]

**supplementary material**

**Literature retrieval strategies**

| **Databases or web page** | **Search strategy** |
| --- | --- |
| CNKI | #1: Topic: (stress ulcer) + (gastric mucosal lesions) or (gastric mucosal erosion) + (gastric mucosa injury) + (stress related mucosal disease) + (gastrointestinal bleeding) + (gastrointestinal hemorrhage)  #2: Topic: (proton pump inhibitors) + (histamine H2 antagonists) + (sucralfate) + (enteral nutrition) + antacids  #3: Topic: (guideline) + (systematic review) + (meta analysis) + (consensus) + ( best evidence)  #4: #1 AND #2 AND #3 |
| Wanfang Data | #1: Topic: (stress ulcer) OR (gastric mucosal lesions) OR (gastric mucosal erosion) OR (gastric mucosa injury) OR (stress related mucosal disease) OR (gastrointestinal bleeding) OR (gastrointestinal hemorrhage)  #2: Topic: (proton pump inhibitors) OR (histamine H2 antagonists) OR (sucralfate) OR (enteral nutrition) OR antacids  #3: Topic: (guideline) OR (systematic review) OR (meta analysis) OR (consensus) OR ( best evidence)  #4: #1 AND #2 AND #3 |
| VIP | #1: M= (stress ulcer) OR (gastric mucosal lesions) OR (gastric mucosal erosion) OR (gastric mucosa injury) OR (stress related mucosal disease) OR (gastrointestinal bleeding) OR (gastrointestinal hemorrhage)  #2: M= (proton pump inhibitors) OR (histamine H2 antagonists) OR (sucralfate) OR (enteral nutrition) OR antacids  #3: M= (guideline) OR (systematic review) OR (meta analysis) OR (consensus) OR ( best evidence)  #4: #1 AND #2 AND #3 |
| Sinomed | ((stress ulcer) OR (gastric mucosal lesions) OR (gastric mucosal erosion) OR (gastric mucosa injury) OR (stress related mucosal disease) OR (gastrointestinal bleeding) OR (gastrointestinal hemorrhage)) AND ((proton pump inhibitors) OR (histamine H2 antagonists) OR (sucralfate) OR (enteral nutrition) OR antacids) AND ((guideline) OR (systematic review) OR (meta analysis) OR (consensus) OR ( best evidence)) |
| Chinese Medical Journals Full-text Database | (stress ulcer) or (gastric mucosal lesions) or (gastric mucosal erosion) or (gastric mucosa injury) or (stress related mucosal disease) or (gastrointestinal bleeding) or (gastrointestinal hemorrhage) |
| Web of Science | #1: TS=((stress ulcer) or (gastric mucosal lesions) or (gastric mucosal erosion) or (gastric mucosa injury) or (stress related mucosal disease) or (gastrointestinal bleeding) or (gastrointestinal hemorrhage))  #2: TS=((proton pump inhibitors) or (histamine H2 antagonists) or (sucralfate) or (enteral nutrition) or antacids)  #3: TS=((guideline) or (systematic review) or (meta analysis) or (consensus) or ( best evidence))  #4: #1 AND #2 AND #3 |
| Cochrane Library | ((stress ulcer) or (gastric mucosal lesions) or (gastric mucosal erosion) or (gastric mucosa injury) or (stress related mucosal disease) or (gastrointestinal bleeding) or (gastrointestinal hemorrhage)) [Title Abstract Keyword] AND ((proton pump inhibitors) or (histamine H2 antagonists) or (sucralfate) or (enteral nutrition) or antacids) [Title Abstract Keyword] |
| CINAHL | ((stress ulcer) or (gastric mucosal lesions) or (gastric mucosal erosion) or (gastric mucosa injury) or (stress related mucosal disease) or (gastrointestinal bleeding) or (gastrointestinal hemorrhage)) [Title] AND ((proton pump inhibitors) or (histamine H2 antagonists) or (sucralfate) or (enteral nutrition) or antacids) [Title] |
| Guidelines and Society Websites | stress ulcer |
